# Complete Genome Sequence of *Staphylococcus aureus* Strain M1, a Unique t024-ST8-IVa Danish Methicillin-Resistant *S. aureus* Clone

**DOI:** 10.1128/genomeA.00336-13

**Published:** 2013-06-06

**Authors:** Hanna Larner-Svensson, Peder Worning, Mette D. Bartels, Lars Hestbjerg Hansen, Kit Boye, Henrik Westh

**Affiliations:** MRSA Knowledge Center, Department of Clinical Microbiology, Hvidovre University Hospital, Hvidovre, Denmarka; Department of Biology, University of Copenhagen, Copenhagen, Denmarkb; Faculty of Medicine and Health Sciences, University of Copenhagen, Copenhagen, Denmarkc

## Abstract

We report the genome sequence, in five contigs, of a methicillin-resistant *Staphylococcus aureus* isolate designated M1. This clinical isolate was from the index patient of a methicillin-resistant *Staphylococcus aureus* (MRSA) outbreak in Copenhagen, Denmark, that started in 2003. This strain is sequence type 8 (ST8), *spa* type t024, and staphylococcal cassette chromosome *mec* element (SCC*mec*) type IVa.

## GENOME ANNOUNCEMENT

The incidence of methicillin-resistant *Staphylococcus aureus* (MRSA) in Denmark is one of the lowest in Europe. However, in 2003 an outbreak of a *spa* type t024, sequence type 8, staphylococcal cassette chromosome *mec* element (SCC*mec*) type IVa (t024-ST8-IVa) MRSA clone started and was not fully under control until 2009. Initially this outbreak was maintained in a closed setting within one of Copenhagen’s boroughs, Amager. The clone spread through the local hospital to the surrounding nursing homes, affecting 501 patients and staff as of the end of 2012 (1–3). This outbreak is now being fully investigated through whole-genome sequencing (WGS).

In order to be able to trace transmission between patients, a complete outbreak-specific reference genome is essential. The reference isolate designated M1 is the isolate from the first patient identified with this MRSA clone in 2003.

The WGS of *S. aureus* strain M1 was determined by high-throughput sequencing, combining Roche GS FLX (Roche Diagnostics, Basel, Switzerland) and Illumina HiSeq (Illumina, CA) sequencing technologies, producing approximately 40× and 760× coverage of the genome, respectively. The GS FLX reads were assembled using Newbler 2.3, resulting in 54 contiguous sequences (contigs) totaling 2.848 Mbp. A comparison of the Newbler and a separate MIRA 3.0 assembly reduced the total number of contigs to 30, plus one closed circular plasmid. Assembly studies showed 8 copies of plasmid per cell. Ten gaps in the bacterial genome were closed by Sanger sequencing of amplification products (ABI 3730; Applied Biosystems, CA). The resulting 13 contigs were oriented and ordered relative to the USA300 TCH1516 sequence, and the order was confirmed by optical mapping (OpGen, Inc, Maryland).

Nine of the remaining 13 gaps were closed through WGS of 96 closely related outbreak isolates, including M1, on an Illumina HiSeq2000. Each isolate was sequenced to an average coverage of 700×. The paired HiSeq reads were assembled using Velvet version 1.1.05. These 96 WGS were blasted to the 13 contigs of the M1 sequence, which made it possible to close all gaps except four of five ribosomal clusters. These ribosomal clusters are almost identical, based on their 5-fold higher coverage than the genome.

Genome annotation was performed automatically on the Rapid Annotation using Subsystem Technology (RAST) server 4.0 (http://rast.nmpdr.org). The genome of *S. aureus* strain M1 consists of a single circular 2,843,075-bp chromosome with 32.8% GC content and one circular plasmid of 27,465 bp. A total of 2,711 coding regions, 56 tRNA genes, 5 rRNA loci, and an SCC*mec* IVa cassette of 27,380 bp (2) were detected.

The WGS of M1 confirms that t024-ST8-IVa is related to other sequenced *S. aureus* genomes, notably USA300 TCH1415 and USA300 FPR3757. M1 shares 2.764 Mb with USA300 TCH1415, corresponding to 99.8% nucleotide identity. A total of 1,107 single nucleotide polymorphisms (SNPs) differentiate the two genomes. M1 has two copies of the prophage Sa2usa and one copy of Sa3usa and is Panton-Valentine leukocidin (PVL) negative and arginine catabolic mobile element (ACME) positive (2, 4).

Further analysis of the genome is now under way to identify factors that might explain the emergence of this MRSA strain in the health care community.

### Nucleotide sequence accession numbers.

The genome data have been deposited in GenBank with accession number HF937103 for the chromosome and HF937104 for the plasmid.
